# Is food addiction a valid and useful concept?

**DOI:** 10.1111/j.1467-789X.2012.01046.x

**Published:** 2012-10-12

**Authors:** H Ziauddeen, P C Fletcher

**Affiliations:** 1 Department of Psychiatry, University of CambridgeCambridge, UK; 2 Metabolic Research Laboratories, Institute of Metabolic Science, University of CambridgeUK; 3 Cambridgeshire and Peterborough Foundation NHS TrustCambridge, UK

**Keywords:** Addiction, binge eating, obesity

## Abstract

In this paper, we consider the concept of food addiction from a clinical and neuroscientific perspective. Food addiction has an established and growing currency in the context of models of overeating and obesity, and its acceptance shapes debate and research. However, we argue that the evidence for its existence in humans is actually rather limited and, in addition, there are fundamental theoretical difficulties that require consideration.

We therefore review food addiction as a phenotypic description, one that is based on overlap between certain eating behaviours and substance dependence. To begin, we consider limitations in the general application of this concept to obesity. We share the widely held view that such a broad perspective is not sustainable and consider a more focused view: that it underlies particular eating patterns, notably binge eating. However, even with this more specific focus, there are still problems. Validation of food addiction at the neurobiological level is absolutely critical, but there are inconsistencies in the evidence from humans suggesting that caution should be exercised in accepting food addiction as a valid concept. We argue the current evidence is preliminary and suggest directions for future work that may provide more useful tests of the concept.

## Introduction

The concept of food addiction (FA) attracts much scientific and popular media interest. Yet, there is a persistent debate over its validity. This is an important debate to hold and resolve because of the potential role of FA in the obesity epidemic. While the idea has intuitive clinical and scientific appeal, and may provide an explanatory narrative for individuals struggling with weight and diet control, it has acquired much currency with relatively little supporting evidence. Despite continuing uncertainty about the concept and relative lack of support, it has remarkable, and, in our view, unjustified, influence in developing neurobiological models of obesity (1) and in framing debates about the formulation of public health policy (2,3). In this paper, we explored the theoretical and empirical foundations for FA and questioned this influence.

We and others have previously examined the neuroscientific (4), behavioural and clinical evidence (5,6) for the addiction model. We will briefly summarize this evidence here. At the outset, it is important to state that we share with many others the view that FA is unlikely to be a causal pathway in the majority of people with obesity, which is a highly heterogenous syndrome. Indeed, an examination of the possible routes to obesity makes clear that an addiction model has a limited, if any, place in understanding obesity (4,7). Although arguments have been made that certain aspects of eating in obesity are ‘addictive’ (8,9), we would caution against less stringent applications of an addiction model as these risk losing the explanatory power and the neurobiological grounding of the model (1). Further, they run the risk of erroneously attributing mechanisms and neural circuitry to observed behaviours. Therefore, we go on to focus on the possible validity of a FA model in the context of a subgroup of individuals in whom obesity is prevalent: specifically those who suffer from binge eating disorder (BED) (10–12). In BED, we have a phenotype that goes beyond obesity with a behavioural profile of disordered and compulsive eating, and this is critical to begin an evaluation of the underlying processes and neural circuitry. Our aim here was to examine the extent to which this model may be more useful in this narrower context and consider what further work would be required to validate it.

## What is addictive?

Before we can begin to answer, or even pose, the question of whether FA is a valid clinical entity, there are some preludial questions that should be considered. The general view expressed in the literature is clear that FA is similar to substance addictions, rather than behavioural addictions like pathological gambling, in that, there is an agent that has a neurochemical effect(s) in the brain. This presumably necessitates the presence of a clearly identifiable addictive agent. While work on animals certainly supports the argument that the combination of high fat and high sugar, prevalent in modern processed foods, produces an addiction-like phenomenon in rodents (13), the FA concept in humans often rests on a less well-explored extrapolation: namely that certain highly processed foods are addictive (2,14). Existing models cannot yet go beyond relating addiction to broad categories of high-fat and high-sugar or hyperpalatable foods, and there are no current ideas about a particular concentration of nutrient(s) that might engender the addictive process. While, of course, a good case can be made for these classes of food being harmful to health from a metabolic and cardiovascular standpoint, this does not help the definition of an addictive substance. We believe that a necessary prelude to examining the FA concept is to recognize three important current limitations to our understanding of what might constitute an addictive food. First, if we intend to examine the model and its neurobehavioural components, it would be important to categorize precisely what this critical addictive element is. Second, as we know from substance addictions, drugs vary in their potency and addictive potential (even within a class of substance), this being partly reflected in their legal classification (15). When we speak of FA, are we talking about many addictive substances or one common substance (fat? sugar?) that drives the addiction across many foods? Third, of those who use drugs, the percentage of individuals who go on to become dependent varies and is small for the majority of drugs (16). The hyperpalatable foods that are thought to be addictive are widely available and widely consumed. To consider that they may become addictive in some individuals will require the characterization of a specific feature (or several features) of these foods that acts in concert with certain individual vulnerabilities.

We do not believe that sufficiently satisfactory progress has yet been made in answering the questions that these uncertainties pose. Be that as it may, the clinical literature on FA has nevertheless advanced quickly in recent years (12,17), supported by growing numbers of neuroimaging studies aiming to draw together aspects of the clinical phenotype of obesity and the underlying neurobiology (see (4) for a review). We see this as an especially positive step given that FA, to be a valid concept, must surely bear some resemblance to drug addiction in terms of neural changes. But, so far, attempts to make the link have been hampered by inconsistency across studies (4). We examine this more closely in the following sections, beginning with an overview of the clinical phenotype and how it is generally used.

## Identifying and measuring food addiction: problems with phenotypic markers

The prevailing phenotypic model of FA is based on similarities between certain aspects of overeating and the Diagnostic and Statistical Manual of Mental Disorders, fourth edition (DSM-IV) criteria for substance addiction (9,18). This similarity has been formalized in the Yale Food Addiction Scale (YFAS) (19), a measure that is forming a cornerstone of the human literature on FA. Devising this scale has necessitated confronting a number of difficulties posed by the fact that, first, food, unlike drugs, is consumed ubiquitously and does not have a simple direct pharmacological action. Therefore, its use and misuse cannot easily be quantified, nor can one identify features of its consumption that indicate a clear transition from use to abuse/addiction. Moreover, certain useful indicators of substance dependence, such as tolerance, withdrawal and expenditure of effort to acquire the addictive substance, require careful thought when translated to the food domain. In obviating these difficulties, the design of the YFAS has had to adopt certain adaptations that have their own limitations. For example, given that there is, as we have discussed, no universally agreed evidence of an addictive agent and that eating behaviour is necessarily part of a continuum, the scale does not have the benefit of being able to dichotomize (is an addictive agent used – yes or no?). It must instead apply severity thresholds and an overall impairment criterion (i.e. food-related behaviour causes significant distress or impairment) in order to distinguish between someone who is addicted and someone who is not. Likewise, with respect to withdrawal symptoms, the scale enquires about ‘anxiety, agitation or withdrawal symptoms …’, but the latter are not, and cannot yet be, clearly defined.

The YFAS was developed with the aim of identifying and quantifying a specific clinical phenotypic entity. A score of ≥3 with the impairment criterion (shown earlier) satisfied is required for the diagnosis of FA. However the score has also been used as a continuous severity measure in individuals who do not endorse sufficient criteria for the diagnosis (see (20)) although it is not clear if there is evidence to support this implied continuum.

The YFAS is undoubtedly an important research tool; however, it does not follow that the syndrome it captures is necessarily FA. It is likely, although, that individuals who endorse the YFAS criteria for FA have a behavioural phenotype with significantly disordered eating behaviour. Whether this is sufficient to define a FA syndrome is debatable.

It is worth pointing out to some important points regarding tolerance and withdrawal. Although these are important considerations in clinical drug dependence, it is recognized that they are not necessarily core elements of the syndrome (21,22), representing, rather, features that indicate prolonged consumption with psychological and physiological adaptations. Indeed, it is a criticism of the DSM-IV criteria for substance dependence that they aggregate core features, such as maintained use despite negative consequences, with markers of long-term use such as tolerance and severity of impairment, e.g. effort spent in acquiring substance. Tolerance and withdrawal relate strongly to the mechanistic action of the addictive substance. Further, they highlight a crucial aspect that has not been very prominent thus far in the FA literature: substance addiction is a disorder with a natural history and course and a set of vulnerability or risk factors. If we are to consider that FA is a disorder then it would need to be similarly characterized.

Before we move on, it would be worthwhile to consider briefly a related and more nuanced view that draws another parallel with substance use disorders: the possibility of food abuse or misuse, i.e. harmful use that is maladaptive, but does not meet the criteria for dependence. Substance abuse is characterized by recurrent use of the substance with one or more of the following features: failure to fulfil role obligations, use in harmful situations, consequent legal problems and persistent use despite negative consequences (23). Given that the behaviours in the food context are part of a continuum of consumption behaviour, one could posit the existence of a food abuse syndrome either as an intermediate stage before the transition to FA or as a less severe pattern of disordered eating. It is our view that such an exploration will become crucial in characterizing the natural history and the neural basis of FA. That is, a close scrutiny of the transitions from use to abuse to addiction will be critical in elucidating the development of the syndrome. However, the merest glance at the criteria for substance abuse makes it clear that translating these criteria to food will pose similar problems to those encountered with the FA model. This brings us to a final concern about a phenotype-based definition of FA: the clinical syndrome of substance addiction may not be the best framework to characterize FA. Perhaps, the way forward might be to outline a more precise neurobehavioural syndrome in which a core set of measurable behaviours is clearly defined (inability to control consumption, increased motivation to consume and persistent consumption despite negative consequences (21,22)). This would capture a range of problem-eating behaviours, including, but not restricted to, binge eating.

In considering the link with obesity, FA may be a cause, a comorbidity or possibly a consequence of obesity and may therefore prevail in non-obese and not-yet-obese individuals. This is not to say that obesity is not a potential surrogate marker of the syndrome if one bears in mind individual vulnerability, and the duration and severity of weight gain. However, it does seem that, as has been argued, BED is a more fruitful area for further exploration of FA as, by definition, it includes an abnormal compulsive eating behaviour that is causing significant impairment and distress. It also has a strong association with obesity (24,25). We, therefore focus on BED and this narrower application of the FA model.

## Narrowing the focus: binge eating

More recent work on FA has focussed on an association with BED (10–12). This condition is classified as an eating disorder in the DSM-IV and is characterized by recurrent episodes (‘binges’) of uncontrolled, often rapid consumption of large amounts of food, usually in isolation, even in the absence of hunger. This eating persists despite physical discomfort and binges are associated with marked distress and feelings of guilt and disgust. Binges can be triggered by negative mood states which are not necessarily ameliorated by the binge (26). An important caveat is that, although BED is associated with obesity, a substantial number of people who show binge eating behaviour are not obese and most obese people do not have BED (25). This observation emphasizes the importance of avoiding simple use of body mass index (BMI) as a general marker for compulsive overconsumption and addiction-like behavior. Using the YFAS, Davis *et al*. found a high comorbidity of FA with BED (72% of people with FA-satisfied criteria for BED compared with 24% of those without FA) as well as greater tendency towards impulsivity and hedonic eating in a sample of 72 obese individuals (12). It should be noted, although, that only 18 people in the sample qualified for a diagnosis of FA. Gearhardt *et al*. (11) showed that 56.8% of a sample of 81 people with BED met YFAS criteria for FA (of some concern is the finding that 54.9% of the sample endorsed withdrawal symptoms, despite the lack of clarity on how they are defined. This it not a minor consideration as participants may well have very different views on what constitutes a ‘withdrawal symptom’). An interesting point to note is that the sample examined by Gearhardt *et al*. had a mean age of 47 and a mean BMI of 40.58 across all the study participants, compared with a mean age of 33.58 and mean BMI of 38.48 in Davis *et al*.'s sample. Taking into account the aforementioned caveats about the measurement instrument and the differing sample characteristics, there is a suggestion that more convincing addiction-like behaviours may be more common in older individuals with higher BMI, as one might predict in a disorder that develops and becomes more severe with time. These data highlight the importance of considering the natural history of this condition and contrasting it with BED.

These points notwithstanding, further observations may support a suggested link between BED and FA. For example, BED has also been associated with polymorphisms of the OPRM1 mu-opioid receptor gene (A118G) and DRD2 dopamine receptor gene (Taq1A A1), both implicated in substance addiction, perhaps suggesting that the genetic vulnerability to this condition may relate to enhanced hedonic eating and a greater drive towards food (27). It does seem that, in exploring FA further, individuals with BED may represent the best target population to study. There is however, a nosological precedence to be cleared up: does one phenomenon subsume the other? That is, do we consider BED to arise because someone has become addicted to food? Or, conversely, does the addiction emerge as a consequence of BED? Of course, these questions are likely to be gross simplifications of a complex relationship and, given the figures identified by Gearhardt *et al*., that 56.8% of people with BED show FA, the overlap is only partial and the conditions/behaviours are dissociable. Critical to further study would be clarifying the phenotype and the natural history of FA in order to determine if it truly is a separate disorder and not merely a set of features, to which the YFAS is sensitive, that prevail in a subgroup of individuals with obesity and BED.

## Moving beyond phenotypic overlap

To summarize the argument so far, an FA may be relevant to a subgroup of individuals with obesity. Many obese people show no signs of behaviours and experiences that would be predicted by an FA phenomenon and while a more useful subgroup to study are those with BED, it is also true that not everyone with BED satisfies criteria for FA and vice versa. Clinical markers only take us so far towards identifying FA and establishing its relationship with existing clinical constructs and categories of eating disorder. Such difficulties can be overcome through well-powered studies recruiting and assessing appropriate diagnostic subgroupings. However, there is a more pressing problem: a prior need to validate the concept of FA itself. It is insufficient to surmise, because some people score highly on the YFAS, that FA is necessarily a valid and unitary concept. A scale cannot simultaneously measure a behaviour and validate a pathophysiological process thought to underlie that behaviour. To achieve any such validation, it seems to us, one must go beyond superficial phenotypic overlap and determine whether the neural changes that co-occur with people appearing to show FA are comparable with those found in more established addictions. This can be done in several ways. The prevalent approach so far has been to assess broadly whether the same sorts of circuitry disrupted in substance addiction are also altered in obesity and binge eating. However, as we have previously asserted (4), this has produced little consensus and has, overall, placed us in the very unsatisfying position of debating whether the evidence is so inconsistent that we cannot accept the existence of FA, or so preliminary that we cannot reject it (10,28). We suggest therefore, that a theoretically more powerful perspective will come from using fuller, process-specific models, based largely on animal neuroscience, in which we consider the process of addiction in terms of precise and dynamic neural and behavioural features that must be characterized longitudinally using correspondingly precise tools from cognitive neuroscience, In the next section, we consider such a theoretically driven approach in more detail.

## A neuroscientific model of food addiction

If, for the sake of discussion, we accept that FA exists (temporarily setting aside the aforementioned concerns) and resembles drug addiction, what predictions would follow from this neuroscientific model?

It would be useful to review briefly the neuroscience of substance addiction. Seminal models of drug dependence have characterized a set of core processes involved in the transition from drug taking to drug dependence. As part of this transition goal-directed drug taking, under ventral striatal and prefrontal control, becomes habitual and compulsive drug-seeking begins to prevail, driven predominantly by the dorsal striatum, with loss of executive control over this behaviour (22). Initially, acute administration of the drug of abuse produces a rise in accumbens dopamine. There is subsequent sensitization of the mesolimbic dopaminergic systems, leading to an enhanced salience of, and consequent motivation towards, drug-related cues (29). However the accumbens dopamine response becomes blunted with the development of addiction and it is instead drug-related cues that produce dopamine increases accompanied by strong, perhaps overwhelming, drug cravings. This has been framed as an enhancement of anticipatory reward with a decrease in consummatory reward. There are also associated impairments in the prefrontal cortex (enhanced salience and compulsivity), dorsolateral prefrontal and inferior frontal cortices (decreased executive control), key areas that connect with the striatum (30).

The development of addiction has also been associated with a decrease in striatal D2 receptors (31), a finding that has been linked to a reward deficiency syndrome (32), where greater levels of drug are taken to produce the same level of reward. However, this view is partly at odds with a model of transition to habitual drug taking, which becomes insensitive to the actual value of the reward. That is, the argument that enhanced drug use emerges as a compensation for reduced consummatory pleasure does not sit neatly with the observations that habitual responses are insensitive to the consequences of consumption. Nevertheless, increasing drug intake leads to neural adaptations in the striatum (further decrease of D2 receptors) that exacerbate compulsive drug-seeking and impaired inhibitory control (31), and in the amygdala that counter the negative states of dysphoria and withdrawal (33). These adaptations serve to perpetuate the syndrome and Koob has described this as the ‘dark side of addiction’ where substance use continues to stave off dysphoria and withdrawal. Interestingly, trait impulsivity, which relates to lower levels of striatal D2 dopamine receptors, has been shown to increase the vulnerability to making the transition to habitual drug taking at least for stimulant drugs (34). The OPRM1 (35,36) and DRD2 genes (37–40) have been implicated in addictions. As mentioned earlier, these genes and the trait of impulsivity have been associated with BED (27). A cannabinoid CB1 receptor polymorphism CNR1 has also been associated with substance use (41) and obesity (42) but not BED *per se*.

It is perhaps worth mentioning that the earlier summary touches on different models of substance addiction that are not entirely complementary and this is worth bearing in mind when extending these findings from the substance addictions models to FA. With regard to an addiction model for food, the following predictions have been made: we would expect to see an enhanced striatal response to food cues and a blunted response to the consumption of actual food rewards. It is not clear what particular cues would be relevant and it is likely that they would be quite individualized. The model is also not sufficiently precisely specified to make predictions about the impact of current state (e.g. hungry or sated) so it is worth mentioning in passing that it seems increasingly likely that careful, individually customized studies would be required. One would also predict that there would be a shift to a greater dorsal striatal role with the development of habitual eating (again, a careful specification of individual variations in the nature, duration and magnitude of altered eating would be necessary). Concomitantly, impairments would be seen in prefrontal, dorsolateral and inferior frontal cortex activity in relation to food cues with associated compulsivity and impaired inhibitory control. D2 receptor levels in the striatum would decrease as part of the neural adaptation to increased consumption, with the development of a negative anhedonic state. Genotypes such as OPRM1 and DRD2 Taq1A polymorphism may determine individual vulnerabilities to these processes.

With this perspective in mind, we consider the evidence thus far for the FA syndrome beginning with the animal literature, which provides the strongest evidence so far.

## Animal models of food addiction

By far, the most convincing evidence for an FA model comes from animal models where rodents exposed to high-sugar, high-fat and a combination of high-sugar high-fat (cafeteria) diets develop behaviours that resemble addiction. These behaviours comprise binge eating, compulsive food-seeking and withdrawal symptoms (13,43). They are accompanied by concomitant neural changes: elevated self-stimulation thresholds, lower striatal D2 receptors (suggesting an anhedonic state) (13) as well as decreased accumbens dopamine (44) and elevated acetylcholine, which are perhaps features of withdrawal (45,46). In sugar addiction models, an opiate-mediated withdrawal syndrome has been demonstrated (46), but this has not been shown for fat or combined high-fat–high-sugar binge eating models (47). The development of compulsive food-seeking resistant to aversive foot shocks (13) is a powerful pointer to the development of compulsivity (22). There is also evidence of an enhanced dopaminergic transmission in the accumbens upon consumption of sucrose (48), but this may be driven by the palatability rather than the nutrient content given that it also occurs with sham feeding of sucrose (49) (see (50)).

Overall, therefore, there are convincing lines of evidence that animals may become addicted to palatable foods. However, there are some important caveats to consider in evaluating the animal data on FA. Animals presented with either high-sugar or high-fat diets, eat excessively, but do not gain weight as they offset the increased intake by eating less chow (43,51). It is only the high fat and sugar combination that causes weight gain (13,52,53). Further, most of these experiments have been conducted in binge eating models, where these changes in behaviour are produced by particular access regimes that do not translate easily to free-living humans. Here, the findings of Kenny and Johnson are particularly salient as in their model, rats had extended access to a cafeteria diet (e.g. bacon, cheesecake) and developed compulsive eating, with escalating consumption and weight gain. These animals also preferentially consumed the cafeteria diet over standard chow. In short, the animal models tell us that it is possible to produce an addiction-like syndrome, one that leads to obesity, with certain nutrient combinations and particular access regimes. These models do validate some of the predictions from the neuroscientific model. However, the findings, while they tell us that hyperpalatable foods, administered in particular, often highly constrained regimens, produce an addiction-like syndrome, they do not afford easy translation to humans who are not subject to such constraints. The most salient conclusion is that behaviour and neural circuitry subserving food reward can be altered by the availability of highly palatable foods in ways that can be compared meaningfully with alterations produced by drugs of abuse. But the question remains: do humans, in their very different environments, become truly addicted to certain nutrients? Here, we turn to the human neuroscience literature: a body of work that will be vital in answering this question.

## The human neuroscience evidence

Unfortunately, the human neuroscience literature is inconsistent and sometimes conflicting (see (4)). Admittedly, there have been only a few studies that have actually explored the neural basis for the FA phenotype, either by characterizing brain regions that correlates with FA behaviours (20) or by examining relevant clinical populations (with, for example, binge eating behaviours (54,55)). Prior to these, a number of studies sought to determine the relationship between brain structure or function and BMI. The earliest evidence came from positron emission tomography (PET) scanning: a seminal study by Wang *et al* (56) showed reduced striatal D2 receptors in individuals with severe obesity and triggered a series of further studies exploring dopaminergic function related to eating and obesity. The earliest work perhaps hinted that the emergent picture would not be straightforward, given a large overlap in receptor levels between obese participants (all with a BMI > 40) and the healthy control group in this study. Subsequently, the finding has been replicated, again with a large overlap between groups, in one study (57), although it should be noted that here, group differences were confounded with state as obese, but not controls were scanned while fasted. Other studies exploring dopamine receptor binding in obesity or binge eating, although they have identified a number of intriguing group differences, including altered responding to pharmacological challenge, have not reproduced this finding and one cannot conclude unequivocally that dopamine receptor levels are altered directly as a consequence or cause of obesity. The same is true of studies exploring functional responses in human reward circuitry, whether to food stimuli, cues predicting food or to pictorial representations of food. We have reviewed these previously (4) concluding that there is little consistent data across these various studies and the findings thus far do not support an addiction model or indeed any one model of altered brain function in obesity. We do not deny that any small selection of findings can be mustered in support of particular variants of the addiction model, but it is hard to get around the fact that the most striking finding is that the between-group differences found across studies are largely conflicting. As most of these studies have phenotyped subjects mainly according to BMI, any interpretation of these data are limited to relationships with BMI alone. Studies exploring within-group variability and relating it to, for example, genetic factors, may offer greater potential for insights into underlying neural causes and consequence of obesity (58). Different predictions from the addiction model have been borne out in some of these studies such as increased striatal and orbitofrontal activation on viewing food images (59,60) or in anticipation of actual food rewards (61), decreased consummatory reward activation (62) and decreased prefrontal metabolism (63) in obese compared with lean individuals. However, once again, these are not consistent findings and no truly coherent picture has yet emerged.

Given the profound limitations in assessing neural changes merely according to BMI, we briefly take a more focussed view of these data from the perspective of an FA model. If we look specifically at studies that have either examined the concept of FA specifically or studied the target group of interest i.e. BED, the literature is far more limited (55). Only one functional magnetic resonance imaging (fMRI) study has looked specifically at people with BED and reported increased orbitofrontal activation on viewing food pictures relative to controls. Similarly, there is one PET study that has examined people with BED and this showed that in these individuals, the combination of methylphenidate and food stimulation reduced dopamine binding in the caudate while this was not seen in non-binge eating obese individuals (54). There has been one study so far that examined FA using the YFAS as the clinical instrument to make the diagnosis. However, none of the subjects in the study met the YFAS criteria for FA and final analyses made an assumption of a continuum, exploring neural responses correlating with YFAS symptom scores. The findings do not support the study's prediction of increased anticipatory and decreased consummatory reward (20).

In summary, the existing neuroimaging literature offers little in the way of support for an FA model and we argue strongly against its selective presentation in support of the FA model, feeling that, ultimately, this will obfuscate a highly complex situation. However, given that there has been little specific exploration of the FA hypothesis, this, as has been argued (10), leaves a very limited dataset with which to draw conclusions about the FA model. But it does suggest that this is a very good time to draw up plans for a systematic exploration of the concept using more precise, theory-led approaches. We consider this in the next section.

## Exploring the neuroscientific evidence for the model: future studies?

In this penultimate section, we consider some further areas for exploration. Two critical questions are the matter of what is addictive and whether the DSM-IV substance dependence is the best framework to study food misuse/abuse/addiction. These questions will require further debate and research, but it should be pragmatic to consider that these concepts may evolve and become clearer with further research into the phenotype and its underlying neurobiology. Integral to these explorations will be longitudinal studies to examine the natural history of the syndrome. Endophenotypic explorations and those focused on symptoms/behaviours may help address the difficulties with characterizing the phenotype. Impulsivity and compulsivity, for example, would be important endophenotypes to consider in the context of an addiction model. Impulsivity may be a key vulnerability factor in obesity and binge eating and a critical one to consider in the development of FA. On the other hand, over the history of the condition one could predict that compulsivity would increase as a function of time, a phenomenon that could either be examined prospectively or correlated retrospectively with duration of illness. Other important factors to consider are reward sensitivity and hedonic eating as well as, crucially, sensitivity to the effects of environmental cues on eating behaviour. To extend further from an addiction model, one could predict that such food-addicted individuals would be more susceptible to the effects of food-related environmental cues than non-addicted individuals. Just as an alcohol binge may arise in response to a subtle and personal cue, so, one imagines, might an eating binge be provoked. Similarly, the relationship with negative emotional states, which are known to trigger binge eating behaviours in BED (26). The role of genotypes such the OPRM1 and the DRD2 Taq1A polymorphism that may mediate these neuropsychological factors will require close scrutiny.

In considering further neuroimaging research, a first step, one that is, no doubt, already being taken, would be to examine a group of individuals who qualify for a diagnosis of FA and examine their brain responses to food with different cognitive challenges to assess the salience of food cues, motivation towards foods and responses to anticipation and consumption of food. These responses could usefully be correlated to measures of symptom severity, compulsivity and craving. Of course, given that the relationship between FA and BED has yet to be fully elucidated (see earlier), careful dissociation of these constructs would be necessary in the interpretation of such work. It is worth noting here that in the Davis *et al*. study that a set of obese non-BED individuals also qualified for a diagnosis of FA. While we agree with the focus on BED, it may be that such non-BED individuals may prove informative in understanding FA and what behaviours the YFAS captures. If we are to examine the neural correlates of FA, it is critical that we define the functional neuroanatomy and neurochemistry of the neural circuit that subserves the processes implicated such as decreased consummatory reward and increased motivation towards food. Pharmacological fMRI could be a useful tool to examine the neurochemistry of the identified circuits both for the purposes to delineating the functional neurochemistry and mechanisms of the process, but also to consider therapeutic strategies. While, understandably, much attention has focussed on the role of dopaminergic and opioidergic systems in an addiction process, it is important to consider the endocannabinoid system. Given the disappointing experiences with the CB1 antagonists (64), it is perhaps unsurprising that the cannabinoid system is not being widely investigated in humans. However the endocannabinoids do have an important role in both hedonic and homeostatic eating (65) and CB1 signalling in the gut enhances fat intake, a mechanism that would be very relevant if high-fat foods are potentially addictive (66). An important consideration with these studies is the modulation of the processes of interest by metabolic factors such as internal states of hunger, adiposity, lean mass and gut hormone levels and variations with BMI.

## Will the food addiction model help treat obesity?

The implications of the addiction model for treatment of obesity and BED are discussed elegantly, and in detail, by Wilson, particularly with regards to psychological treatment (5). The rather damning conclusion with respect to the FA construct, is that successful therapeutic approaches to treatment of, for example, binge eating, are quite different to what would be proposed were the condition to be meaningfully explained by an addictive process. With regards to pharmacological treatment, at present the question is moot as there is little in the way of effective pharmacological treatment for addictions or obesity. Mu-opioid dysregulation has been implicated in binge eating and mu-opioid antagonists such as naltrexone have been trialled for the treatment of binge eating with very limited success (67). However, this is a very important consideration as, if FA is to have any clinical value it must add something to the treatment of sufferers either in terms of developing/selecting the appropriate psychological therapy or the right pharmacological treatment. Although it may be far too premature to consider it seriously at present, the possibility of OPRM1 and DRD2 variants facilitating pharmacogenetic approaches to treatment, may well merit exploration.

## Conclusion

This paper was written to contribute to a brief and, one hopes, helpful debate about FA – the evidence for and against its validity and its usefulness as a construct in taking us forward at a time when altered patterns of human consumption pose a major and global threat to health. We believe that the debate, which goes well beyond the papers presented here, is at a sufficiently mature stage to obviate the need for simplistic and dichotomized positions. While our starting point is that any reasonably comprehensive review must conclude that FA is a rough and incomplete descriptive phenomenon that is unsupported by existing evidence, such a perspective represents a starting point rather than a conclusion. We have therefore sought to be more positive, trying to suggest some ways in which the model could be explored further with a view to determining its validity. We take very seriously a recent caution against ‘tossing the baby out with the bathwater’ (10) by simply dismissing the concept before the appropriate neuroscientific studies have been done in humans. However, we reiterate that partial and selective views of the existing literature invoked to support the model, no matter how conceptually compelling that model may seem, will be a profound hindrance. We further argue against wider and less stringent applications of the model to obesity as a whole and emphasize that it is very important that an addiction model add something valuable to the understanding and treatment of obesity.

Before we conclude, we would like to step outside the field of neuroscientific examination to the wider societal context. It is important to consider why this model has gathered such momentum in the field and in the media. It seems quite intuitive that the model does offer some solace to individuals struggling with eating and weight and does offer a counterweight to a prevalent view of obesity as a moral failing on the part of the obese individual. Certainly, there has been associated (and valid) criticism of fast food companies for encouraging excessive consumption and a movement to encourage greater industrial responsibility in food manufacture, such as the ‘Responsibility deal’ in the United Kingdom (although neither of these specifically relate to FA). While this is laudable, given that there is, at present, insufficient evidence to support the notion of FA, it is of some concern that the scientific community has been suggesting that FA mandates the modification of public health policy in much the same way that nicotine addiction did for smoking (2). While we are happy to concede that the evidence is too preliminary to reject the concept of FA (10), it follows that such a state of affairs counsels strongly against the use of such an untested notion in attempts to guide policy making.

However, looking ahead, it is worth giving some consideration to the ideas that are being suggested for policy change such as restrictions on high-fat and high-sugar foods. It will be intriguing to see the effects of natural ‘experiments’ being proposed such as bans on large drinks in New York or those already underway like the fat tax in Denmark. We should be mindful of the valuable lessons from the world of substance addiction. The classifications of drugs of abuse (and therefore the attendant legal ramifications) are periodically reviewed, not necessarily based on scientific evidence alone (as societal value judgements play a significant role (68)). It is salutary to remember that, in such case, the addictive agents are already clear, in contrast to the case with food. Enforcing the relevant legislation is not always straightforward with drugs that are clearly identified and is likely to be far more problematic with foods. While it is difficult to imagine the idea of an illegal cheesecake dealer it is not too difficult to consider the problems that may arise in restricting some foods from some people/groups and not others. We conclude on this cautious note, highlighting that even if FA were to be validated as a disorder, there is much further to go to make it clinically useful and the eagerly proposed formulation of public health policy around such a model would be quite complicated. Perhaps, ultimately, the scientific endeavour will be best directed towards the development of an evidence base that could guide the formulation of legislation relevant to food industry practices.
